# Cholesterol-Lowering Bioactive Foods and Nutraceuticals in Pediatrics: Clinical Evidence of Efficacy and Safety

**DOI:** 10.3390/nu16101526

**Published:** 2024-05-18

**Authors:** Federica Fogacci, Naif Saad ALGhasab, Valentina Di Micoli, Marina Giovannini, Arrigo Francesco Giuseppe Cicero

**Affiliations:** 1 Hypertension and Cardiovascular Risk Factors Research Center, Medical and Surgical Sciences Department, Sant’Orsola-Malpighi University Hospital, Via Albertoni 15, 40138 Bologna, Italy; federica.fogacci@studio.unibo.it (F.F.); valentina.dimicoli2@unibo.it (V.D.M.); marina.giovannini3@unibo.it (M.G.); 2 Department of Internal Medicine, Medical College, Ha’il University, Ha’il 55476, Saudi Arabia; 3 Department of Cardiology, Libin Cardiovascular Institute, Calgary University, Calgary, AB T2N 1N4, Canada; 4 Cardiovascular Medicine Unit, Heart, Thoracic and Vascular Department, IRCCS Azienda Ospedaliero-Universitaria di Bologna, 40138 Bologna, Italy

**Keywords:** nutraceuticals, functional foods, dietary supplements, dyslipidemia, hypercholesterolemia

## Abstract

Long-term exposure to even slightly elevated plasma cholesterol levels significantly increases the risk of developing cardiovascular disease. The latest evidence recommends an improvement in plasma lipid levels, even in children who are not affected by severe hypercholesterolemia. The risk–benefit profile of pharmacological treatments in pediatric patients with moderate dyslipidemia is uncertain, and several cholesterol-lowering nutraceuticals have been recently tested. In this context, the available randomized clinical trials are small, short-term and mainly tested different types of fibers, plant sterols/stanols, standardized extracts of red yeast rice, polyunsaturated fatty acids, soy derivatives, and some probiotics. In children with dyslipidemia, nutraceuticals can improve lipid profile in the context of an adequate, well-balanced diet combined with regular physical activity. Of course, they should not be considered an alternative to conventional lipid-lowering drugs when necessary.

## 1. Introduction

Optimal low-density lipoprotein cholesterol and/or triglyceride plasma levels significantly protect against cardiovascular disease (CVD) development, and higher levels are associated with an increased risk [1,2]. The optimal values are defined based on age and ethnicity. Dyslipidemias are largely prevalent disorders of lipoprotein metabolism that can result in abnormal lipid and lipoprotein values. The prevalence of dyslipidaemias is increasing all over the world, even in Mediterranean countries where the diet should be qualitatively healthier. A recent Italian study showed a prevalence of suboptimal cholesterolemia (total cholesterol–TC > 170 mg/dL) in 78% of children and adolescents [3]. Recent studies suggest that long-term mild exposure during childhood to even a mild suboptimal LDL-C level is associated with an increased risk of coronary artery disease. International guidelines suggest screening children and adolescents for lipid levels, especially in families with dyslipidemia and/or atherosclerotic CVD (ASCVD) [4,5].

In a large Australian cohort, subjects who had incident non-high density lipoprotein-cholesterol (non-HDL-C) dyslipidemia from childhood to adulthood and those with persistent dyslipidemia had dramatic increased risks of cardiovascular events (Hazard Ratio 2.17 [95% Confidence Interval (CI) 1.00–4.69] and Hazard Ratio 5.17 [95% CI 2.80–9.56], respectively), when compared with those whose non-HDL-C levels remained within the guideline-recommended range in childhood and adulthood. Then, participants who had high non-HDL-C in childhood but whose non-HDL-C levels were within the guideline-recommended range in adulthood did not have a significantly increased risk (Hazard Ratio 1.13 (95%CI 0.50–2.56)) [6].

Thus, improvements in physical activity and some dietary behaviors that ensure all necessary nutrients for adequate growth during childhood are advisable [7,8], although specific lipid-lowering pharmacological treatment is needed to manage secondary and severe genetic dyslipidemias [9,10]. Some nutraceuticals have clearly demonstrated cholesterol-lowering activity in adults. Experts recommend their utilization in managing individuals who have a low estimated risk of developing ASCVD, but who show an insufficient metabolic response to dietary changes. Additionally, nutraceuticals may be considered for some low-risk patients with statin-intolerance when combined with ezetimibe [11,12].

According to the American Academy of Pediatrics (AAP) and European Atherosclerosis Society (EAS), the first step in the management of adult and pediatric patients with hypercholesterolemia is nutritional approach and life-style management, followed by drug therapy [5,13].

Functional foods or “nutraceuticals” have been used as adjunct treatment for adult patients, and are suggested for pediatric patients with familial hypercholesterolaemia (FH) [13].

Studies on cholesterol-lowering nutraceuticals in children and adolescents are relatively limited, as only a few short-term, randomized, controlled studies have been performed. These are mainly concerned with the administration of (soluble) fibers and plant sterols/stanols, whereas sporadic reports have tested the efficacy and tolerability of standardized red yeast rice extract, soy proteins, probiotics, and Omega-3 and Omrega-6 polyunsaturated fatty acids (PUFAs).

This review article aimed to critically summarize the scientific evidence supporting cholesterol-lowering dietary supplements and nutraceuticals in managing children and adolescents with dyslipidemia.

We focused on the most widely used nutraceuticals in dyslipidemia management, especially those that have shown evidence of modifying plasma levels of LDL-C and triglycerides. Additionally, we also examined the impact of these nutraceuticals on TC and HDL-C levels, to provide clinicians comprehensive objective information to guide their decision-making process.

## 2. Materials and Methods

A detailed literature search was performed in this narrative review on PubMed using the following keywords: “Children”, “Adolescent”, “Pediatric”, “Hypercholesterolemia”, “Hypertriglyceridemia”, “Dyslipidemia”, “Dietary supplement”, “Nutraceutical”, “Efficacy”, “Tolerability”, “Safety”, “Cholesterol-lowering” and “Lipid-lowering”. Preference was given to placebo-controlled randomized clinical trials. The collected articles underwent independent review by two authors. Inclusion criteria were established prior to article review, and were as follows: (i) all published scientific papers that describe dietary supplement, nutraceuticals and lipid-lowering agents in pediatric population; and (ii) high-quality systematic research, randomized control trials, study cohorts and cross-sectional studies that were published in English. We excluded studies that were not reported in English, and those that exclusively focused on adults (age ≥ 19 years).

Findings were classified by the main mechanisms of action (i.e., cholesterol absorption inhibitors from the bowel, LDL synthesis inhibition by the liver, and a mixed mechanism of action), and summarized in tables and figures. The review tables included nutraceutical, study type, study aim, participants, intervention, intolerance, compliance and observed effects.

## 3. Natural Cholesterol Absorption Inhibitors from the Bowel

Several natural compounds can interfere with cholesterol absorption, significantly improving cholesterolemia in children and adolescents.

### 3.1. Soluble Fibers

Fibers are edible parts of plants that pass through the small intestine relatively unchanged in humans, and include complex carbohydrates such as non-starch polysaccharides (pectins, gums, cellulose, hemicellulose, oat bran and wheat bran), oligosaccharides (inulin and fructooligosaccharides), and lignin (i.e., the non-carbohydrate fraction of the dietary fiber). Fibers intake is linked to positive health outcomes, including a lower risk of developing ASCVD and obesity [14]. Fibers naturally contained in cereals, vegetables and fruits (as part of a balanced dietary pattern) ameliorate the lipid profile in adults [15], and reduce concentrations of TC and Low Density Lipoprotein-Cholesterol (LDL-C) by 5–15% and 9–22%, respectively [16,17]. These beneficial effects have led regulatory agencies to issue health claims for the intake of fibers [18], oat β-glucan and its LDL-C lowering effect or ASCVD risk reduction [19,20].

A large cohort study on 5873 Japanese children (10–11 years) highlighted the presence of an inverse association between the consumption of dietary fibers and plasma concentrations of TC, and the presence of overweight and obesity, confirming data from clinical trials in adults [21]. Although dietary fibers help maintain good health, their quantitative need in children has not been defined yet [22]. Food and Drug Administration (FDA) guidelines relate it to the need for calorie intake (12 g/1000 calories), while the American Academy of Pediatrics guidelines relate it to weight or age [23]. Pediatric recommendations widely vary across countries, being also influenced by the available evidence [24]. In general practice, many pediatricians follow a formula that involves adding 5 g/day to the child’s age (for children older than 3 years) [25]. Even if this guideline is often considered when advocating for a Mediterranean diet or a diet rich in vegetables, it is seldom met in clinical practice.

Children living in Western countries typically consume fewer vegetables and fruits, contributing to a lower dietary fiber intake, high calorie dense food, and highly refined high-fat diet [26]. The consequences of such an incorrect dietary intake include metabolic changes and hyperlipidemia, which are sometimes associated with overweight/obesity. In this context, a positive effect of dietary fibers on blood lipids with monounsaturated FAs (MUFAs) was shown in children in the Healthy Start Preschool Study of Cardiovascular Disease Risk Factors and Diet [27].

Soluble fibers include psyllium (viscous and non-fermentable fiber), glucomannan, oat, pectin, and guar gum (viscous and fermentable fiber) and are commercially available as unprocessed fibers that can be added to food or used as flavored powders or capsules. Psyllium, derived from the seed husk of *Plantago ovata*, is one of the richest sources of soluble mucilaginous dietary fiber, acting as a gel-forming polysaccharide, similarly to pectin and guar gum [28]. Locust bean gum (that is, a galactomannan from the carob tree) is a white and odorless powder extracted from the endosperm of beans without a distinctive taste. Pectin is less viscous than other fibers, but similar in ash and more palatable [29]. Glucomannan, the main polysaccharide obtained from the Asian tuber Amorphophallus konjac, is a palatable, highly viscous soluble fiber. Its chemical structure consists of a mannose (8): glucose (5) ratio linked by b-glycosidic bonds. Glucomannan has the highest molecular weight and viscosity among all dietary fibers [30,31]. Oat seeds are also an important source of the viscous soluble fiber beta-glucan [32].

The ability of fibers to lower plasma lipids relies on their physicochemical properties and viscosity. Soluble fiber acts mainly to form viscous solutions that slow gastric emptying and reduce fat absorption, thereby modulating lipoprotein metabolism. In the small intestine, the gelling process binds to dietary fats and hinders the absorption of cholesterol, and the reabsorption of bile acids increases their excretion in feces. It follows that there is a reduced uptake of intestinal cholesterol and a reduced circulation of chylomicrons. Of consequence is that the synthesis of bile in the liver increases and LDL-C levels decrease. Another cholesterol-lowering mechanism involves bacterial fermentation in the colon (except for lignin), which leads to production of short-chain FAs (acetate, propionate, and butyrate). Propionate inhibits cholesterol synthesis [33,34].

A relatively small number of short-term randomized clinical trials have investigated the cholesterol-lowering effect of fibers in children and adolescents with largely variable results, ranging from no effect to a 30% reduction in LDL-C plasma levels. The most frequently studied fiber is psyllium, followed by glucomannan, oats, and gum, which are usually added to STEP I (daily fat intake < 30%, saturated FAs < 10%, cholesterol < 300 mg) or STEP II (saturated FAs 7%, cholesterol < 200 mg), now indicated as CHILD I and CHILD II diet [35,36,37,38,39,40,41,42] (Table 1).

Balancing the fiber intake from food and nutraceuticals or fiber-added food is relevant to compliance and outcomes. A very restricted diet—as required by the STEP II diet—even if safe, is not always well accepted by children [43,44]. Combining the STEP I diet with food-enriched or capsule-containing fiber is often more effective in reducing LDL-C levels.

Many studies have demonstrated the efficacy of psyllium [45,46]. A 12-week randomized controlled study on 50 children with mild hypercholesterolemia on a STEP I diet supplemented with psyllium (3.2 g/daily) showed an additional 8.9% LDL-C decrease compared with the controls [40]. Further, in 36 children with familial combined hyperlipoproteinemia, supplementation with psyllium (2.5–10 g, depending on the age) to a STEP I diet led to a TC and LDL-C level reduction of 11.9% and 13.8%, respectively [34]. Psyllium supplementation also improved the LDL-C lowering effect of the STEP II diet in children with hyperlipidemia [41], different from what was previously observed in a randomized, double-blinded, placebo-controlled, cross-over study employing 6 g/day of psyllium in 20 children with mild hypercholesterolemia, already on the STEP II diet [36].

Glucomannan supplementation was successfully tested in 36 children with hyperlipidemia who underwent a double-blinded, randomized, placebo-controlled cross-over trial that lasted 24 weeks. This cohort, affected by primary dyslipidemia, was fed a CHILD I diet for ≥1 month. Capsules containing glucomannan (500 mg) were administered at a dose of 1000–1500 mg/day depending on the proband’s weight. TC, LDL-C, and non-HDL-C levels decreased significantly by 5.1%, 7.3%, and 7.2%, respectively [47]. Consistent with previous findings, these results were more pronounced in females than males [41]. However, two meta-analyses of the LDL-C-lowering effect of glucomannan supplementation in children did not confirm any positive effect of this fiber on LDL-C levels [48,49].

**Table 1:** Main characteristics of the available clinical studies testing the cholesterol-lowering effects of different fiber types in children.

Nutraceuticals	Type of Study [Reference]	Primary Aim of the Study	Participants	Main Inclusion Criteria of the Study	Intervention	Follow-Up	Intolerance and/or Side Effects	Compliance	Main Effects of the Tested Nutraceutical on LDL-C	Main Effects of the Tested Nutraceutical on Other Lipid Fractions
PSYLLIUM	Randomized, double-blind, crossover, controlled clinical trial [35]	To assess the LDL-C lowering effect of psyllium-enriched breakfast cereal in children with hyperlipidemia	N. 32 children (6–18 years)	LDL-C > 90th percentile for age and sex at baseline	58 g of a psyllium-enriched cereal/day for a total daily dose of 6.4 g soluble fiber from psyllium or placebo without psyllium	6 weeks	N. 1 child experienced gastrointestinal effects with slight abdominal bloating	Good compliance	−6.8%	↓ TC (−5%). No change in HDL-C and TG.
	Randomized, double-blind, placebo-controlled clinical trial [41]	To assess the LDL-C-lowering effect of psyllium in children with hypercholesterolemia	N. 20 children (5–17 years)	LDL-C > 110 mg/dL after ≥3 months of a controlled diet	6 g/day ready-to-eat cereals with water-soluble psyllium fiber or placebo without psyllium	4–5 weeks	No side effects	Good compliance (80%)	No change	No change in TC and HDL-C. ↑ TG in the control compared with the psyllium group.
	Randomized, clinical trial [50]	To assess the TC and LDL-C lowering effect of psyllium in children	N. 36 children (3–17 years)	Children with primary type IIa hypercholesterolemia	Children < 7 years: 5 g/day psyllium. Children > 7 years: 10 g/day psyllium.	8 ± 2.4 months	No side effects	Good compliance	−23%	↓ TC (−18%). No changes in HDL-C and TG.
	Randomized, single-blind, placebo-controlled clinical trial [45]	To assess the TC and LDL-C lowering effect of psyllium in children	N. 50 children (2–11 years)	LDL-C ≥ 110 mg/dL	Psyllium-enriched cereal containing 3.2 g of soluble fiber, each box of placebo cereal containing < 0.5 g of soluble fiber	12 weeks	No side effects	Good Compliance	−15.7%	↓ TC (−9.6%). ↑ HDL-C (+9.96%). ↓ TC/HDL ratio (−17.9%). ↓ LDL/HDL ratio (−21.1%).
	Randomized, double-blind, placebo-controlled clinical trial [46]	To assess the LDL-C lowering effect of psyllium in Brazilian children and adolescents with mild-to-moderate dyslipidemia	N. 49 children (6–19 years)	TC > 4.40 mmol/L	7.0 g/day psyllium or 7.0 g/day cellulose (placebo)	8 weeks	No side effects	N. 2 children in the control group were excluded during follow up	−10.7%	↓ TC (−7.7%). No change in HDL-C and LDL-C/HDL-C ratio.
GUM	Randomized, crossover, controlled clinical trial [29]	To assess the LDL-C lowering effect of locust bean gum	N. 17 adults and N. 11 children (N. 18 participants with FCH + N. 10 controls) (Adults: 22–53 years; children: 10–18 years)	One parent and one child and ≥1 additional first-degree relative with FCH; Families with increased incidence of premature ASCVD.	Children: 10–20 g/day locust bean gum; Male Adults: 25–35 g/day locust bean gum; Female Adults: 10–25 g/day locust bean gum.		No side effects	Good compliance in adults	Participants with FCH: ○ −11% Participants with FCH ○ −19%	Participants with FCH: ↓ TC (−10%). Participants with FCH: ↓ TC (−17%).
GLUCOMANNAN	Randomized, double-blind, crossover, placebo-controlled clinical trial [47]	To assess the efficacy and tolerability of dietary supplementation with glucomannan in children	N. 36 children (6–15 years)	TC levels higher than the age- and sex-specific 90th percentile	2/week capsule containing either 500 mg of glucomannan or placebo	8 weeks	No side effects	Good compliance (92% compliance for the dietary supplement and 90% compliance for the placebo)	−7.3%	↓ TC (−5.1%). ↓ non–HDL-C (−7.2%). No significant change in TG, HDL-C, Apo-AI, and Apo-B.
PECTIN	Non randomized, controlled clinical trial [36]	To assess the efficacy of wheat bran and pectin mix on plasma lipids in children	N. 51 children (4–18 years) with high LDL-C and N. 33 controls (5–16 years)	LDL-C serum levels > 135 mg/dL after 6 months of dietary counselling (for the actively treated group)	Dietary counselling and dietary supplementation with 50% wheat bran + 50% of pectin, 2–3 tablets/day (50 mg × Kg/day)	3 months	N. 2 children experienced abdominal discomfort and soft stools	Good compliance	−17%	↓ TC (−15%). ↓ TG (−18%). ↓ ApoB (−14%). No significant change in HDL-C and ApoA-1 levels.
GLUCOMANNAN + POLICOSANOLS OR CHROMIUM POLYNICOTINATE	Randomized, double-blind, placebo-controlled clinical trial [51]	To assess the effect of low-dose chromium-polynicotinate (Group A) or policosanols (Group B), and their glucomannan combination (Group C and Group D, respectively) in children	N. 120 children (3–16 years; Male, N. 60–Female: N. 60)	TC > 170 mg/dL. No drug treatment before enrollment. No dietary treatment in the previous three months. One parent with TC > 240 mg/dL or a family history of ASCVD in first- or second-degree relatives.	Group A: 0.2 mg Chromium polynicotinate + 500 mg Starch 500 mg. Group B: 1.2 mg Policosanols + 500 mg starch. Group C: 500 mg Glucomannan 500 mg + 0.2 mg Chromium polynicotinate. Group D: 500 mg Glucomannan + 1.2 mg Policosanols. Group E: 500 mg Glucomannan + 500 mg starch. Children ≤ 6 years: 2 capsules each at lunch and dinner; children > 6 years: 3 capsules each at lunch and dinner	8 weeks	No side effects	N. 6 children refused to follow the diet N. 6 children refused to take capsules	Group B: ○ No effect Group C: ○ −16% Group D: ○ −10%	Group B: ○ No lipid-lowering effects. Group C: ○ No change in HDL-C and TG. Group D: ○ No change in HDL-C and TG.

↑, Increase; ↓, decrease; Apo-B, apolipoprotein B; Apo-A1, apolipoprotein A1, HDL-C, high-density lipoprotein cholesterol; LDL-C, low-density lipoprotein cholesterol; TG, triglycerides; TC, total cholesterol; FH, familial hypercholesterolemia; FCH, familial combined hyperlipidemia; N., number.

Among other fibers, oat bran supplementation has been tested in children in several clinical trials [52]. For instance, oat bran significantly increased HDL-C levels and reduced LDL-C levels after 7 months of consumption (dosage: 1 g/kg body weight/day) compared with soy derivatives in 20 children with hypercholesterolemia (5–12 years) [53].

Furthermore, locust bean gum (Carruba) showed a significant 11–19% LDL-C level decrease when comparing active and placebo groups in a 16-week cross-over controlled trial, including 11 children with familial combined hyperlipidemia, 10 controls, and 17 adults who consumed locust bean gum (8–30 g/daily) [54].

Among these interventions (Table 1), psyllium consistently showed the highest reduction in LDL-C levels, ranging from 6.8% to 23%. This was followed by gum interventions, which resulted in a reduction of LDL-C levels ranging from 11% to 19%. Pectin interventions demonstrated a significant reduction of 17% in LDL-C levels. Lastly, Glucomannan interventions combined with Chromium polynicotinate or policosanols showed moderate reductions, ranging from 7.3% to 16% in LDL-C levels. It is important to note that these interventions were effective in lowering LDL-C levels in pediatric populations, but the extent of reduction varied across studies. The compliance was overall good, even if some children refused to follow the prescribed diet or take the capsules.

Even if a fiber rich diet is always to be preferred, when the intake is not sufficient, supplemented fibers are usually safe and well tolerated. Mild intestinal discomfort has been reported in clinical trials.

### 3.2. Plant Sterols

Plant sterols, also known as phytosterols or non-cholesterol sterols, are natural compounds found in plants, and are commonly consumed through foods like vegetable oils and nuts. These compounds are ingested in amounts comparable to cholesterol intake (200–400 mg/day), which cannot be synthesized by the human body. Plant sterols effectively and safely lower serum cholesterol levels by hindering cholesterol absorption [55]. Since 2001, plant sterol-enriched foods have been recommended by the National Cholesterol Education Program Guidelines as part of dietary strategies to reduce LDL-C levels [56].

Non-cholesterol sterols, or stanols (in the form of sterol esters), are available commercially, and are added to various foods such as bread, cereals, salad dressings, milk, margarine, and yogurt, often with different flavors and a good taste [57]. Studies have shown that, in adults, incorporating stanols into the milk matrix yielded better results compared to cereals, with LDL-C levels decreasing by 15.9% versus 5.4%, respectively [58]. While stanols were more effective in reducing cholesterol levels compared to sterols, most studies administered sterols at varying doses ranging from 1.6 to 2 g daily.

**Figure 1:**
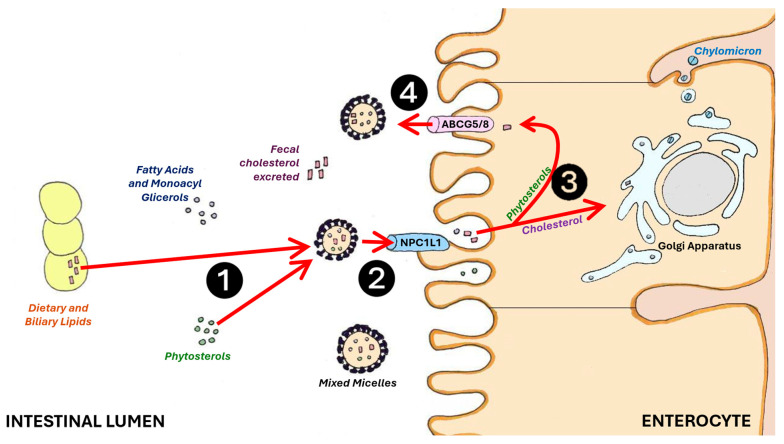
Phytosterols (❶) compete with cholesterol absorption at the intestinal brush border membrane by reversibly binding the NPC1L1 protein (❷). Free phytosterols (❸) are excreted into the intestinal lumen by ABCG5/8 in the intestinal cells (❹), and enhance cholesterol and bile salts excretion with feces (red arrows). ABCG5/8: adenosine triphosphate-binding cassette transporter G5/8; NPC1L1: Niemann–Pick C1-Like 1.

Plant sterols work by inhibiting cholesterol absorption in the intestines, leading to a reduction in serum cholesterol concentration [59]. Phytosterols, particularly sitostanol, compete with cholesterol for absorption in the intestines and displace cholesterol from micelles (Figure 1).

Phytosterols are more hydrophobic than cholesterol, making them more susceptible to mixed micelles. Cholesterol and phytosterols rely on Niemann–Pick C1-Like 1 (NPC1L1) protein for absorption into enterocytes. Once absorbed, non-esterified cholesterol and phytosterols are transported back into the intestinal lumen through the action of the ABCG5/G8 gene. Approximately 50% of the cholesterol, but less than 5% of plant sterols, is ultimately absorbed [60,61]. Phytosterols, when in their free form, are absorbed at low rates (less than 10%), while stanols are not absorbed physiologically [62]. The reduced uptake of intestinal cholesterol and its transport via chylomicrons to the liver result in decreased levels of intermediate-density lipoproteins in addition to LDL-C [63].

In several randomized clinical trials, plant sterols significantly decreased cholesterolemia in children with mild hypercholesterolemia and FH [63,64,65,66,67,68,69,70,71,72] (Table 2).

Dietary supplementation with 1.2–2.0 g/day sterols has been mainly tested in children with FH who had already been on STEP I or II, showing a further LDL-C lowering effect of ~10% in 2–12 months [63,65,67]. In children with FH, a daily intake of 2.3 g phytosterols significantly decreased TC (−11%) and LDL-C (−14%) levels compared with placebo spread [69], whereas higher decreases were observed in children undergoing stanol-added diet (3 g/day) [63]. Apolipoprotein B (Apo-B) levels were also significantly reduced by plant sterols (7–10%) [63,65,69]. The efficacy of phytosterols was further demonstrated in non-FH children on a STEP II diet and with mild hypercholesterolemia (mean TC > 197 mg and LDL-C > 125 mg/dL). The daily intake of 1.2 g plant sterol in two doses reduced TC (from −7% to −11%) and LDL-C (from −9% to −14%) levels, respectively, compared with the control group [64].

Margarine containing 1.6 g/day plant sterols or plant stanol ester reduced TC (−9%) and LDL-C (−12%) in children with FH after for 5–6 weeks [66,68], while in the STRIP study, 6-year-olds with mild hypercholesterolemia significantly decreased TC and LDL-C, respectively, by −5.4% and −7.5% [60]. Then, plant sterol supplementation could safely reduce LDL-C by roughly 10% and without significantly affecting other lipoprotein levels [73]. Remarkably, the Apo E4 or E3 genotypes were not reported to influence the biochemical effects of sterol addiction in children [65]. The administration of milk, yogurt and margarine frequently could influence the cholesterol-lowering effect of phytosterols [57,74], whereas the lipid drop seems independent of baseline levels, being the maximum effect usually reached in a short time (2 weeks, usually) [75].

An additive benefit of the above-mentioned changes is the significant decrease in small dense LDL-C levels after the daily dietary supplementation of 2 g plant sterols in children and adolescents with dyslipidemia [68]. It must, however, be recognized that TG and HDL-C concentrations in plasma are usually unaffected by phytosterol supplementation [76,77], as well as the endothelial function [72].

Children undergoing statin therapy show homeostatic changes characterized by increased cholesterol absorption and plant sterol levels. Phytosterol supplementation reverses these changes, and should be considered advantageous [78,79].

Phytosterols are usually safe and well-tolerated. Variations in carotenoids and fat-soluble vitamins have been reported by studies that used plant sterol- or stanol ester-enriched spreads in adults and children. In children with FH, lipid-adjusted lycopene levels decreased by 8.1% (*p* = 0.015) during the stanol period; however, this reduction was not significant at the 6-month follow-up. In addition, alfa- and beta-carotene levels significantly decreased by 17.4% and 10.9%, respectively, in children with FH after the daily consumption of 1.2 g plant sterols for 2 months, recovering at the 6-month follow-up [64]. In the Special Turku Coronary Risk Factor Intervention Project for children (STRIP study), the dietary supplementation of 1.5 g phytosterols in children with mild hypercholesterolemia was associated to a decrease (−19%; *p* = 0.003) in serum beta-carotene to LDL-C ratio, while the alpha-tocopherol to LDL-C ratio remained unchanged [60]. Moreover, no changes were observed in the levels of the other carotenoids or fat-soluble vitamins [66]. To the extent that there is little data, improving vegetables and fruits intake in children should be suggested as add-on to phytosterol-added dietary regimen, to compensate for any possible reduction in carotenoid also related to seasonal dietary variations.

Long-term safety was questioned as phytosterol plasma levels increased the incidence of atherosclerosis [80], and should be related to an increased risk of cardiovascular events, as described in the large epidemiological cohorts of the PROCAM and MONICA/KORA studies [81,82]. Premature atherosclerosis has also been observed in the rare autosomal recessive familial form of sitosterolemia [83]. However, campesterol and sitosterol under physiological conditions do not exceed 1% of the total serum sterols, whereas cholesterol accounts for >99% of serum sterols. Moreover, lathosterol was not modified over a 12-week period, proving that the inhibition of cholesterol absorption by phytosterols does not cause an increased cholesterol synthesis [70]. 

### 3.3. Probiotics

Probiotics have a limited evidence of cholesterol-lowering effects in adults. This effect results from cholesterol absorption and bile salts hydrolysis (BSH) [84,85]. The first mechanism, activated by lactic acid bacteria, suppresses the reabsorption of cholesterol in the intestines, while the second mechanism affects the balance of bile salts, resulting in a decrease in plasma LDL-C levels. Moreover, certain strains of bifidobacteria improve blood lipid levels by converting linoleic acid (LA) into conjugated linoleic acid (CLA) [86].

A recent umbrella systematic review of 38 meta-analyses concluded that the probiotics supplementation was effective in reducing TC (effect size [ES], −0.46 mg/dL; 95%CI, −0.61, −0.30; *p* < 0.001), TG (ES, −0.13 mg/dL; 95%CI, −0.23, −0.04; *p* = 0.006), and LDL-C levels (ES, −0.29 mg/dL; 95%CI, −0.40, −0.19; *p* < 0.001), without affecting HDL-C [87] The evidence in children is rare. A 32-week-long, double-blinded, randomized, placebo-controlled, cross-over trial was conducted involving children whose TC levels exceeded the 90th percentile for their age and sex. Administering a mixture of three bifidobacterium strains, selected for characteristics that ameliorated the lipid profile, such as BSH activity, cholesterol adsorption, and CLA production, mildly but significantly improved TC (3.4%), LDL-C (3.8%), and TG (1.9%) levels, and increased HDL-C (1.7%) levels [88]. The effect seems less impressive than that in adults; however, the effect could depend on the tested probiotic formulation, as the probiotic action depends on the strain, strain mix, dosage, and administration medium. Probiotic supplementation is usually safe [89] and well-tolerated. The available clinical data regarding probiotics in adults is inconclusive, and there is limited information available regarding their effects in children. Therefore, it would be premature to definitively state that probiotics have a significant lipid-lowering effect, given the limited evidence available.

## 4. Cholesterol-Lowering Dietary Components, and Nutraceuticals Acting through Different Mechanisms

Other dietary and food components with cholesterol-lowering actions beyond inhibiting cholesterol absorption from the bowel have also been tested in children and/or adolescents. These trials were usually small, short-term, and limited to specific settings (Table 3), suggesting the need for further confirming larger and long-term studies.

### 4.1. Nuts

Nuts (i.e., almonds, hazelnuts, pistachios, walnuts, macadamia nuts and peanuts) are classified as dry fruits. In recent decades, the cardioprotective and health-promoting qualities of nuts—particularly walnuts—have been extensively demonstrated in epidemiological studies. These positive effects are due to their composition rich in bioactives, such as monounsaturated fatty acids (MUFAs), polyunsaturated fatty acids (PUFAs), tocopherols (vitamin E), antioxidants, phytosterols, fibers and polyphenols, with hazelnuts being especially noteworthy in this regard [97]. Additionally, hazelnuts stand out for having the highest content of MUFAs among all nuts [98]. Regular hazelnut intake also seems to improve microbiota composition and reduce the intestinal concentration of short-chain fatty acids (SCFAs) [96]. Moreover, polyphenols influence cholesterol absorption, TG synthesis and secretion and exert antioxidant effects [99].

To the best of our knowledge, only a few small trials until now have investigated the effects of hazelnuts on plasma lipids in children. Hazelnuts peeled or with the skin (0.43 g/kg of body weight, 15–30 g portions) were shown to significantly reduce LDL-C, while the ratio HDL-C/LDL-C increased compared to a control group receiving the STEP I diet. Moreover, their intake increased the MUFA/saturated fatty acids (SFA) ratio in red blood cells and lowered the endogenous and oxidative induced DNA damage [98].

### 4.2. Soy

**Figure 2:**
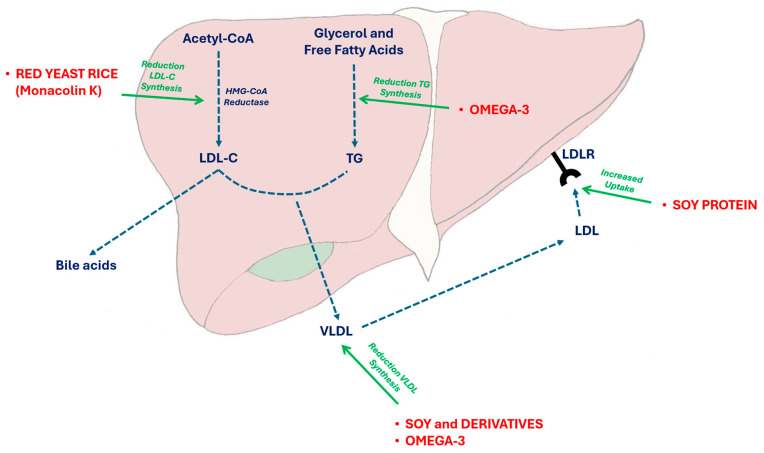
Effects of some nutraceuticals on triglyceride and cholesterol synthesis. VLDL liver secretion and LDL uptake. In particular, red yeast rice is able to reversibly inhibit the HMGCoA reductase activity, while soy proteins main act by increasing the LDL-C receptor on liver cell surface and Omega 3 PUFAs by inhibiting the Apolipoprotein CIII and the lipoprotein-lipase activity. Acetyl-CoA: acetyl-coenzyme A; HMGCoA: 3-hydroxy-3-methylglutaryl-coenzyme A; LDL: low-density lipoprotein; LDL-C: low-density lipoprotein cholesterol; LDLR: low-density lipoprotein receptor; TG: triglycerides; VLDL: very-low-density lipoprotein cholesterol.

Soy contains several bioactive compounds that are supposed to improve plasma lipid levels in humans (i.e., isoflavones, phytosterols and specific peptides, which can promote LDL-C receptor expression in liver cells) (Figure 2). 

A recent meta-analysis showed that a median intake of 25 g/day of soy proteins during a median follow-up of 6 weeks decreased LDL-C plasma levels by 3–4% in adults (−4.8 mg/dL; 95%CI −6.7, −2.8 mg/dL, *p* < 0.0001) [95]. However, only few studies involving children exist. In a pilot study testing the cholesterol-lowering effect of soy or milk protein in children with FH, TC and LDL-C levels did not significantly change though TG and HDL-C improved [91]. Different results were obtained in a prospective study conducted on 16 children with FH. TC, LDL-C, and ApoB significantly improved (−7.7%, −6.4% and −12.6%, respectively) after a 3-month period in which soy proteins were incorporated into the Step I diet, being administered in the form of soy-based dairy-free milk at a dosage of 0.25–0.5 g/kg body weight [92]. This study represents an extended examination of soy protein in pediatric populations. Interestingly, even if children were generally adherent to the program, not all participants (4 out of 16) exhibited the desired response, despite having similar characteristics at entry. Overall, most studies concur on the efficacy of soy protein; however, safety concerns—such as allergic reactions or the potential effects of dietary phytoestrogens (i.e., isoflavones)—remain debatable, especially in the youngest participants [100]. A 13-week randomized controlled clinical trial has been recently launched to address the effect of a soy-rich diet compared to a low-fat diet and a control diet in children with FH. After 7 weeks from randomization, the reduction in LDL-C levels was notably greater in the soy group (155 ± 29 mg/dL) compared to the control group (176 ± 28 mg/dL; P for comparison = 0.038), with a similar trend observed at 13-week follow-up (LDL-C = 180 ± 42 mg/dL in the control group and 155 ± 30 mg/dL in the soy group; P for comparison = 0.089). The relative decrease in LDL-C levels was significantly associated with plasma isoflavone concentrations (specifically daidzein and genistein), as measured at week 7 [101]. Moreover, it must be acknowledged that soy proteins are usually well tolerated, and the occurrence of acute reactions depends on the individual hypersensitivity. However, total protein intake must be balanced with soy intake, and safety of the phytoestrogens must be confirmed on the long term.

### 4.3. Polyunsaturated Fatty Acids (PUFAs)

The dietary fats composition is a crucial determinant of lipid concentrations in plasma [102]. Nonetheless, the dietary intake of polyunsaturated fatty acids (PUFAs)—including Omega-3 and Omega-6—is frequently insufficient in children [103]. 

Dietary supplementation of PUFAs have shown to impact cardiovascular risk markers, such as TG, LDL-C and adhesion molecules, while also possessing anti-inflammatory properties [104,105]. In a randomized double-blinded placebo-controlled clinical trial involving 107 healthy children, PUFAs and low saturated fatty acids were able to significantly reduce markers of endothelial cell activation (i.e., adhesion molecules, E-selectin, ICAM-1 and lymphocyte levels), while increasing plasma concentration of Docosahexaenoic acid (DHA) after receiving a 5-month daily intake of a milk enriched product [106].

Another functional food that has been studied in this context is the hempseed oil, which is notably rich in essential fatty acids, including Omega-3 PUFA α-linolenic acid (ALA) and Omega-6 PUFA linoleic acid (LA), with an LA/ALA ratio ranging between 2:1 and 3:1 [95]. The cholesterol-lowering effect of hempseed oil has been extensively assessed in animals and adult humans. However, a recent 8-week randomized controlled trial showed that HSO increases the content of total n-3 and n-6 PUFAs in red blood cells and improves the Omega-3 index, even in children with hyperlipidemia. Moreover, according to the findings of the study, hempseed oil exerted significant reductions in LDL-C (−14%) compared to the control [94]. Overall, emerging observations offer valuable insights for enhancing and complementing food intake with PUFAs. However, further studies are warranted to validate preliminary data.

### 4.4. Red Yeast Rice

Red yeast rice is a widely used and clinically tested cholesterol-lowering nutraceutical derived from the fermentation of standard rice by specific mycelia (usually *Monascus purpureus* Went) with the production of a pigment (making the rice red) and some bioactive compounds, among which monacolins are reversible inhibitors of 3-hydroxy-3-methyl-glutaril Coenzyme A reductase (Figure 2) [107]. The most bioactive compound was monacolin K, which is chemically analogous to lovastatin. In adults, monacolin K significantly reduces cholesterolemia [108] while maintaining an acceptable safety profile [109]. To date, only one study has been conducted in children at increased cardiovascular risk, including those with familial hypercholesterolemia (FH) and familial combined hyperlipidemia, while following a step II diet [90]. The study, designed as a double-blinded, randomized cross-over trial, spanned 6 months in total, during which all participants completed the trial without experiencing any notable adverse effects. After 8 weeks of treatment, there was a significant reduction in TC, LDL-C, and ApoB levels by −18.5%, −25.1%, and −25.3%, respectively, while HDL-C remained unchanged. These findings were remarkable in terms of compliance, tolerability, and efficacy. Notably, the administered dose of Monacolin K was 3 mg/day, demonstrating impressive results comparable to those achieved with pravastatin (10 mg/day or more), indicating a potential synergistic effect with other bioactive components. A recent change in European Union (EU) regulations forbids the use of red yeast rice in children and adolescents because of the presumed (but not demonstrated) risk to health [110].

## 5. Discussion

Multiple nutraceuticals have shown efficacy in reducing lipid levels, as evidenced by the available clinical trials. However, it is important to note that no single dietary supplement can replace the importance of proper dietary counseling. Lifestyle changes and adherence to a correct Mediterranean diet showed a mean decrease of 9.5% in TC, 13.5% in LDL-C, and −10.9% in non-HDL-C plasma levels in a recent large retrospective study conducted on children with polygenic and familial hypercholestremia [111]. This may be sufficient to manage mild polygenic hypercholesterolemia.

Phytosterols and fiber-enriched foods are usually shown to be effective in reducing TC and LDL-C levels in children with FH or polygenic hypercholesterolemia or children affected by other secondary dyslipidaemias, especially when combined with a STEP I (daily fat intake < 30%, saturated FAs < 10%, cholesterol < 300 mg) or STEP II (saturated FAs 7%, cholesterol < 200 mg) diet. ApoB is also ameliorated after phytosterol intake, while contrasting results were observed after fiber intake. In contrast, no favorable variations have been observed in the endothelial function of phytosterol/sterol addition, and only two studies concerning this topic are inconclusive. Plant sterol/stanol and fiber were well received, with high compliance observed, particularly in the short term. Some gastrointestinal side effects would be expected if phytosterols are assumed with addition of artificial sweeteners. Noteworthy is the absence of significant adverse effects; nonetheless, abdominal discomfort or diarrhea were commonly reported symptoms with fiber supplementation. They could be even more frequent and severe when fibers are assumed with addition of artificial sweeteners. Functional foods incorporating phytosterols/stanols were associated with a notable decline in carotenoids, but not other vitamins, highlighting the importance of maintaining an adequate intake of vegetables and fruits to prevent nutritional deficiencies.

While randomized and controlled studies focusing on robust endpoints in children are lacking and infrequent, certain benefits have been noted, particularly in the short term, primarily relating to plant sterol/stanol and fibers. Many other bioactive compounds have demonstrated efficacy as cholesterol-lowering agents in adults (red yeast rice, berberine, bergamot polyphenol fraction, and artichoke extracts), but not in children. Presently, no dietary supplements have been shown to significantly reduce lipoprotein (a) levels, beyond L-carnitine and Coenzyme Q10, but this effect has never tested in children [112].

The European Atherosclerosis Society Consensus Panel recommended that functional foods containing phytosterols to be considered for children with familial hypercholesterolemia, and as dietary additive supplementation rather than independent pharmaceutical treatment, especially with lifestyle modification [113,114]. Despite the limitation on the available evidence of dyslipidemia in pediatric age group, guidelines and evidence suggest that nutraceuticals, particularly fibers and phytosterols, can be utilized when combined with appropriate diet in children with genetic dyslipidemias starting from the age of 6 years old [10,115]. However, it could make sense if the treatment with cholesterol-lowering nutraceuticals are adequately dosed, long-term and effective.

## 6. Conclusions

Lifestyle and dietary modifications are recommended for any child or adolescent presenting with mild to moderate dyslipidemia. Phytosterols and fibers are deemed safe, while the other mentioned nutraceuticals may serve as possible efficacious additions to dietary treatment when combined with appropriate diet regimen. It is important to note that nutraceuticals should not be viewed as substitutes for diet or statins when they are medically indicated, as advised by the main international guidelines.

## Figures and Tables

**Table 2:** Main characteristics of the randomized controlled trials testing the cholesterol-lowering effect of different types of phytosterols/stanols in children.

Type of Study [Reference]	Primary Aim of the Study	Participants	Main Inclusion Criteria of the Study	Active Intervention	Follow-Up	Intolerance and/or Side Effects	Compliance	Main Effect of Dietary Supplementation with Phytosterols/Stanols
Controlled clinical trial [66]	To assess the effect of the daily consumption of 2 g of plant sterols on sdLDL-C levels in children	N. 59 children (4.5–15.9 years; N. 25 with LDL-C ≥ 130 mg/dL and N. 34 with LDL-C < 130 mg/dL)	Not specified	Yogurt enriched with 2 g/day plant sterols	6–12 months	No serious adverse event	Good compliance (86.6%)	↓ sdLDL-C (−16.6%). ↓ LDL-C (−13%). No change in HDL-C, TG and Lp(a).
Double-blind, crossover, controlled clinical trial [62]	To assess the effect of the daily consumption of milk enriched with 1.2 g/day of plant sterol on cholesterol in children	N. 30 children (6–9 years)	TC > 170 mg/dL. LDL-C > 110 mg/dL.	Milk enriched with 1.2 g/day of plant sterol	8 weeks	No serious adverse events. N. 1 child experienced nausea	Good compliance	↓ TC (−4.5%). ↓ LDL-C (−11.1%).
Randomized, double-blind, crossover, controlled clinical trial [69]	To assess the effect of plant stanols on lipids and endothelial function in pre-puberal children	N. 40 children (7–12 years)	Diagnosis of FH by detecting mutation or an LDL-C > 95th percentile for age and sex and one parent with FH	500 mL of a low-fat yogurt enriched with 2.0 g of plant stanols	4 weeks	No serious adverse events	Good compliance (98% during the plant stanol consumption group and 96% in the control group)	↓ TC (−7.5%). ↓ LDL-C (−9.2%). No change in HDL-C, TG and FMD.
Randomized, clinical trial [68]	To assess the efficacy, tolerability, and safety following dietary supplementation with plant sterol in children	N. 52 children (8–16 years)	Outpatients with primary hyperlipidemia (e.g., HeFH, FCH or primary hypercholesterolemia)	Plant sterol-enriched yogurt (100 mL, 1.6–2.0 g/day of sterol)	12 weeks	N. 1 child experienced abdominal discomfort	N. 2 children had poor adherence to the diet program; N. 2 children had difficulties drinking the yogurt	↓ LDL-C levels: ○ −10.7% in children with FH; ○ −14.2% in children with FCH; ○ −16% in children with polygenic hypercholesterolemia. ↓ CT. ↓ ApoB. No change in TG, HDL-C and Apo-A1.
Randomized, crossover, clinical trial [65]	To assess changes in plasma lipids, plant sterols, fat-soluble vitamins and carotenoids in children and parents with FH consuming plant sterol ester-enriched spread	N. 37 children (7–13 years) and their parents (N. 20; 32–51 years)	Diagnosis “definite” or “possible” HeFH (for children and their parents)	In children: Lipid-lowering diet + 13.7 g/days of plant sterol ester-enriched spread (1.2 g plant sterols). In parents: Lipid-lowering diet + 16.5 g/days of PSE spread (1.5 g of plant sterols).	26 weeks	No serious adverse events	Good compliance	↓ TC (−9.1% in children and parents). ↓ LDL-C (−11.4% in children; −11.0% in parents). ↑ Lathosterol. ↑ Campesterol (+32% in children). ↑ Sitosterol (+48% in children). ↓ α- and β-carotene (−17.4% and −10.9%, respectively).
Randomized, double-blind, crossover, placebo-controlled clinical trial [63]	To assess the effects of sex, Apo-E phenotype, cholesterol absorption, and synthesis on the cholesterol-lowering effect of plant stanol esters in children	N. 81 children (6 years)	Children recruited from the STRIP study	Replacement of dietary fat intake with 20 g/day of plant stanol ester margarine	3 months	No serious adverse events	Good compliance	Boys: ○ ↓ TC (−6%). ○ ↓ LDL-C (−9%). Girls: ○ ↓ TC (+4%). ○ ↓ LDL-C (+6%). No sex differences. ↓ Cholesterol absorption in apoE4+ and apoE4−. ↑ Cholesterol synthesis in the apoE4+ group.
Randomized, double-blind, crossover, placebo-controlled clinical trial [64]	To assess the effect of the sterol ester-enriched spread on serum lipids, lipoproteins, carotenoids, fat-soluble vitamins, and physiologic variables in children	N. 38 children (7–12 years)	Diagnosis “definitive” or “possible” HeFH	Child 1 diet + 18.2 ± 1.5 g/day of sterol ester-enriched spread (1.60 ± 0.13 g sterol ester)	8 weeks	No serious adverse events	Good compliance (91.7% in the actively treated group and 90.9% in the control group)	↓ LDL-C (−10.2%). ↓ TC. ↓ ApoB (−7.4%). No changes in HDL-C, TG, and ApoA1. ↓ Lycopene (−8.1%). ↑ Retinol (−15.6%). ↑ α-tocopherol (−7.1%).
Randomized, double-blind, crossover, placebo-controlled clinical trial [67]	To assess whether the ratios of squalene and cholesterol precursor sterols to cholesterol, cholestanol, and plant sterols to cholesterol differently change in the plasma of children after dietary supplementation with stanol esters	N. 23 children (2–9 years)	TC > 194 mg/dL and TG < 176 mg/dL	19.9 g/day of stanol ester-enriched spread (1.6 g stanol esters) and 21 g/day of stanol ester-enriched spread (1.7 g stanol esters)	5 weeks	No serious adverse events	Good compliance	Stanol: ○ ↓ TC (−9%). ○ ↓ LDL-C (−12%). Sterol: ○ ↓ TC (−6%). ○ ↓ LDL-C (−9%). No changes in HDL-C or TG.
Randomized, double-blind, crossover, placebo-controlled clinical trial [70]	Effect of plant sterols on cholesterol and vascular function in prepuberal children with FH	N. 61 children (5–12 years)	LDL-C > 95th percentile for age and sex. Family history of hypercholesterolemia with LDL-C levels > 95th percentile for age and sex before treatment or FH diagnosis by detecting a mutation in the LDL receptor gene.	15 g of plant sterol-enriched spread (2.3 g plant sterols)	4 weeks	No serious adverse events	Good compliance	↓ TC (−11%). ↓ LDL-C (−14%). No change in FMD.
Randomized, double-blind, clinical trial [61]	Effects of sitostanol (3 g/day) ester dissolved in rapeseed oil margarine as a hypocholesterolemic agent in one child with homozygous and 14 children with He-FH maintained on a low cholesterol diet for 6 weeks	N. 15 children with FH (i.e., HeFH and HoFH; 2–15 years)	FH diagnosis established in children and in one of the parents by DNA technique	24 g/day of rapeseed oil-rich margarine with sitostanol ester (mean sitostanol intake of 2.76 ± 0.15 g/day)	6 weeks	No serious adverse events	Good compliance	↓ TC (−10.6%). ↓ IDL (−25.9%). ↓ LDL-C (−15%). ↑ HDL/LDL (+27%). ↑ Δ-cholestenol (+9%). ↑ Lathosterol (+42%). ↑ Desmosterol (+29%).

↑, increase; ↓, decrease; FH, familial hypercholesterolemia; FMD, flow-mediated dilation; He, heterozygous; Ho, homozygous; HDL-C, HDL-cholesterol; IDL, intermediate density lipoproteins; LDL-C, LDL-cholesterol; sdLDL-C, small dense low density lipoprotein-cholesterol; TC, total cholesterol; TG, triglycerides; N., number.

**Table 3:** Main characteristics of randomized controlled trials testing the cholesterol-lowering effect of other nutraceuticals in children.

Nutraceutical	Type Of Study [Reference]	Primary Aim of the Study	Participants	Main Inclusion Criteria of the Study	Intervention	Follow-Up	Intolerance and/or Side Effect	Compliance	Main Observations
RED YEAST RICE EXTRACT AND POLICOSANOLS	Randomized, double-blind, crossover, placebo-controlled clinical trial [90]	To assess the efficacy, tolerability and safety of treatment with a dietary supplement containing red yeast rice extract and policosanols in children	N. 40 children (8–16 years)	Hypercholesterolemia (i.e., FH, FCH or polygenic hypercholesterolemia)	200 mg/day red yeast rice extract (~3 mg/day of monacolins) and 10 mg/day policosanols	8 weeks	N. 3 children experienced headache, diarrhea and dyspepsia. N. 2 children experienced mild CPK increase.	Good compliance	↓ TC (−18.5%). ↓ LDL-C (−25.1%). ↓ Apo-B (−25.3%). No change in HDL-C and Apo-A1.
SOY PROTEINS	Randomized, clinical trial [91]	To assess the effect on serum lipids and lipoproteins of 3-month treatment with a soya-substituted diet in children	N. 16 children (4–18 years)	Diagnosis of FH in children and at least one family member with FH	Diet substituting soy protein (≥0.25 g/Kg body weight) for animal protein	3 months	N. 2 children showed allergy to soya proteins. N. 5 children did not adhere to the study design.	Good compliance	↓ TC (−7.7%). ↓ LDL-C (−6.4%). ↓ ApoB (−12.6%). No change in HDL-C, Apo-A1 and Lp(a).
	Randomized, crossover, controlled, clinical trial [92]	To assess the effect on plasma lipid and lipoprotein levels of a standard low-fat and -cholesterol diet compared with a low-fat and -cholesterol diet substituting soy protein for animal protein	N. 23 children with FH or polygenic hypercholesterolemia (N. 12 male children and N. 11 female children; 9.3 ± 4.5 years)	FH or polygenic hypercholesterolemia	Soy proteins in diet	8 weeks	Not relevant adverse effects	Not assessed	↓ TC (−16%). ↓ LDL-C (−22%).
	Randomized, double-blind, crossover, placebo-controlled clinical trial [93]	To assess the effect on plasma lipoprotein concentrations of soy proteins and cow milk proteins in children	Children	FH	Soy proteins. Cow-milk proteins.	4 weeks	No relevant adverse effects	Not indicated	↓ TG. ↓ VLDL-C. ↑ HDL-C. No changes in TC, LDL-C and apolipoproteins.
PUFA	Randomized, controlled, clinical trial [94]	To assess the effect of dietary supplementation with hempseed oil on plasma lipid profile and FA composition of RBCs in children	N. 36 children	Polygenic hypercholesterolemia and compliance with dietary guidelines	3 g hempseed oil (1.4 g LA and 0.7 g/day ALA)	8 weeks	No relevant adverse effects	Not indicated	↓ RBC SFAs (−5.02%). ↓ RBC MUFAs (−2.12%). ↑ n-3 PUFAs (+1.57%). ↑ n-6 PUFAs (+5.39%). ↑ Omega 3 Index (+1.18%). No changes in plasma lipid profile.
NUTS	Randomized, single-blind, controlled, clinical trial [91]	To assess the effect of dietary intervention with hazelnuts on plasma lipids, anthropometric parameters, and FAs composition of erythrocyte phospholipids	N. 60 children (11.6 years)	Primary hyperlipidemia, with TC and/or TG > 90th percentile	15–20 g/day hazelnuts	8 weeks	No relevant adverse effects	Not indicated	↓ LDL-C (−6.5%). ↑ HDL/LDL (+8%).
	Randomized, controlled, clinical trial [95]	To assess the effect of a dietary intervention with hazelnuts on selected oxidative stress markers in children	N. 60 children	Normal weight. Primary hyperlipemia, with TC level and/or TG > 90th percentile.	15–20 g/day hazelnuts	8 weeks	No relevant adverse effects	Not indicated	↓ endogenous DNA damage (−18.9%). No change in Ox-LDL.
	Randomized, controlled clinical trial [96]	To assess the effects of regular hazelnut intake on microbiota composition and SCFA levels in children	N. 32 children (7–17 years)	Normal weight. Primary hyperlipemia, with TC and/or TG > than age- and sex-specific 90th percentiles.	0.43 g/kg hazelnuts body weight (≤30 g)	8 weeks	No relevant adverse effects	Not indicated	Modulation of fecal levels of predominant intestinal SCFAs.

PH, polygenic hyperlipidemia; LA, linoleic acid; ALA, α-linolenic acid; RBC, red blood cells; FAs, fatty acids; FCH, familial combined hypercholesterolemia; FH, familial hypercholesterolemia; SCFAs, short-chain fatty acids; SFAs, saturated fatty acids; MUFAS, monounsaturated fatty acids; PUFAs, polyunsaturated fatty acids; IME, intestinal microbial ecosystem; LDL-C, low-density lipoprotein cholesterol; N., number; TC, total cholesterol; TG, triglycerides.
